# Modification of the Surface Morphology and Properties of Graphene Oxide and Multi-Walled Carbon Nanotube-Based Polyvinylidene Fluoride Membranes According to Changes in Non-Solvent Temperature

**DOI:** 10.3390/nano11092269

**Published:** 2021-08-31

**Authors:** Jungryeong Chae, Taeuk Lim, Hao Cheng, Wonsuk Jung

**Affiliations:** School of Mechanical Engineering, Chungnam National University, Daejeon 34134, Korea; wndfud486@naver.com (J.C.); taewook9409@g.cnu.ac.kr (T.L.); chenghao@g.cnu.ac.kr (H.C.)

**Keywords:** multi-walled carbon nanotubes (MWCNTs), graphene oxide (GO), polycinylidene fluoride (PVDF), surface morphology, phase separation, coagulation temperature

## Abstract

The effect of changes in non-solvent coagulation bath temperature on surface properties such as morphology and hydrophilicity were investigated in multi-walled carbon nanotubes (MWCNTs) and graphene oxide (GO)-based polyvinylidene fluoride (PVDF) membranes. The properties of pores (size, shape, and number) as well as membrane hydrophilicity were investigated using field emission scanning electron microscopy, Raman spectroscopy, optical microscopy, water contact angle, and water flux. Results showed that the pore size increased with an increase in coagulation temperature. The hydrophilic functional groups of the added carbon materials increased the solvent and non-solvent diffusion rate, which significantly increased the number of pores by 700% as compared to pure PVDF. Additionally, these functional groups changed the hydrophobic properties of pure PVDF into hydrophilic properties.

## 1. Introduction

Separating membranes are widely used in various water treatment studies owing to their high treatment efficiency in a single process and their ability to reliably remove suspended substances over a certain size [1,2,3,4]. Most membrane materials are made of polymers such as polycarbonate [5,6,7], poly(ether sulfone) [8,9,10], polytetrafluorethylene [11,12], and polyvinylidene fluoride (PVDF) [12,13,14,15,16].

PVDF is a fluorine-based polymer material which exhibits high thermal stability, good chemical resistance, and excellent mechanical strength; therefore, it is widely used in the water treatment field [15]. Additionally, it exhibits strong hydrophobicity and low surface energy. Owing to its strong hydrophobicity, the organic matter present in water is easily adsorbed onto the membrane surface or clogged pores, making it prone to low transmittance and fouling. Changing the existing hydrophobic properties to hydrophilic properties is the most effective method of solving this issue. The surface can be modified by adding a hydrophilic substance to the polymer [17]. Therefore, the development of copolymers and methods for introducing hydrophilicity through nanomaterials is being studied extensively.

Graphene oxide (GO) has various oxygen functional groups, including carbonyl, carboxyl, and hydroxyl groups [18,19]. It is suitable for providing hydrophilicity to the membrane and for maintaining stable dispersion in an aqueous solution with oxygen functional groups. Multi-walled carbon nanotubes (MWCNTs) are employed in various fields, such as in sensors and batteries, owing to their high flexibility, low mass density, high porosity, and antibacterial properties [20,21]. Therefore, many efforts have recently been made to introduce nanoparticles into polymer matrix to form nanocomposite membranes that can exhibit superior properties to those of pure polymer membranes. Among various nanomaterials, graphene and its derivatives graphene oxide (GO) and carbon nanotubes (CNTs) are considered as good fillers for the synthesis of nanocomposite membranes. Silva. et al. [22] prepared MWCNT/PVDF composite membranes and investigated different synthesis parameters (loading and surface chemistry of MWCNTs, porosity agent loading, and PVDF molecular weight). Meng. et al. investigated the effect of the dissolving temperature of PVDF in the DMAC/GO solution [23]. However, research on the effects of non-solvent coagulation bath temperature on the MWCNTs/GO/PVDF composite membranes has not been reported.

The filtration performance of the PVDF membrane can be determined by surface characteristics such as permeability, size, shape, and the number of pores. These properties are affected by the materials. They are based on the method of fabrication [24].

The most common membrane manufacturing method is the phase inversion method, which includes non-solvent-induced phase separation (NIPS) [25,26,27,28], thermally-induced phase inversion (TIPS) [25,29], and vapor-induced phase separation (VIPS) [30,31]. The NIPS method has been most widely used to prepare homogeneous solution [28]. This NIPS method consists of three components: the polymer, solvent, and non-solvent. The interaction between the polymer and the solvent is controlled by dissolving the polymer in a solvent [25]. Then, the polymer solution is molded into a specific shape on the substrate and immersed in a non-solvent such as DI water. The solvent and non-solvent mutually change with each other and the film is formed by precipitation of the polymer in a non-solvent bath for solidification [26]. Thereby, through the immersion of a substrate in the solidification bath, the solvent in the cast film solution is exchanged with the non-solvent in the precipitation medium, and phase separation occurs. This process produces asymmetric films with a dense top layer and a porous sublayer [27]. However, the fabrication method varies depending on the type of polymer and the solvent. Additionally, the membrane properties are affected by the type of solvent, temperature, and speed [32,33,34].

In this study, we added GO and MWCNTs to a pure PVDF membrane to investigate the effects of distributed carbon nanomaterials on the membrane properties while changing the coagulation bath temperature using optical microscopy, water contact angle measurement, Raman spectroscopy, field emission scanning electron microscopy (FE-SEM), and water flux measurement. In terms of the results, the hydrophilic functional groups of GO and CNTs changed the hydrophobic properties of pure PVDF to hydrophilic ones, which induced an increase in the number of pores by 700% as compared to pure PVDF. Additionally, the increased coagulation bath temperature accelerated the rate of phase conversion and diffusion rate of the solvent and non-solvent.

## 2. Materials and Fabrication Methods

**Figure 1:**
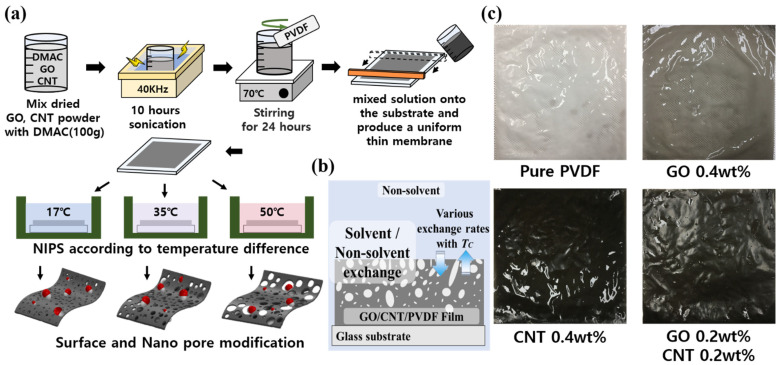
(**a**) The fabrication of GO and MWCNT-based PVDF membranes according to the content ratio of carbon materials and the change in coagulation temperature. (**b**) Conceptual diagram of non-solvent-induced phase separation. (**c**) Optical images of fabricated PVDF membranes with 0.4 wt% GO, 0.4 wt% CNTs and 0.2 wt% GO + 0.2 wt% CNTs, respectively.

Carbon nanomaterial-based PVDF membranes were synthesized based on the NIPS phenomenon, as shown in Figure 1. GO (V-20) was purchased from Standard Graphene, Korea. Carbon nanotubes (CNTs, CM-130) were purchased from Hanwha Chemical, Seoul, Korea. PVDF was purchased from Dongguan Zhanyang Polymer Materials Inc., Guangdong, China. The chemical composition and dimensions such as diameter and length of GO and CNTs used in these experiments are referred to in the Supporting Information.

The GO and MWCNT-based PVDF membrane was manufactured according to the differences in the non-solvent (DI water) temperatures of the conventional NIPS method. The fabrication process is illustrated in Figure 1. Several synthesized ratio conditions were selected to analyze the effects of GO and MWCNTs against the pure PVDF membrane with changes in the coagulation temperature of pure PVDF, GO (0.4 wt%), CNTs (0.4 wt%), and GO (0.2 wt%) + CNTs (0.2 wt%).

GO, CNTs, and PVDF were dried in an oven at 80 °C for 24 h according to their respective composition ratios. The dried GO and CNTs were mixed with dimethylacetamide (DMAC; purity 99.5% purchased from DAEJUNG CHEMICALS & METALS, Shiheung, Korea) and they were dispersed for 10 h by sonication (40 kHz). The dispersed GO/CNTs/DMAC solution was mixed with PVDF by mechanical stirring at 80 °C for 24 h. Subsequently, sonication (2 h, 40 kHz) was conducted to manufacture the casting solution. The manufactured GC–PVDF casting solution (30 mL) was applied to a glass substrate by pushing it with a steel blade to produce a uniform thin GC–PVDF membrane with a thickness of 120 μm ± 0.2 μm as shown in Figure 1. The applied solution and the glass substrate were immersed in deionized (DI) water at different temperatures (17, 35, and 50 °C) to induce other effects of phase transition reaction such as the surface and pore modifications. The casting solution reacted with DI water to cause solidification, and the residual solution was removed to form a membrane. The fabricated membranes with varying GO and MWCNT contents are shown in Figure 1b. The higher GO and CNT contents produced darker PVDF.

We measured the water contact angles of the different PVDF membranes using a 17 μL DI water droplet by the water contact angle measurement equipment (Phoenix-10, Surface Electro Optics, Gyeonggi-do, Korea; See Supporting Information) to observe the surface changes owing to the content of carbon material and the coagulation temperature. Additionally, the Raman spectra of these fabricated membranes with varying GO and MWCNT contents were acquired using a high-resolution dispersive Raman microscope (ARAMIS, HORIBA KOREA Ltd., Anyang-si, Korea) under an excitation wavelength of 532 nm. FE-SEM (Hitachi S-4800, Hitachi High-Technologies Corp., Tokyo, Japan) was employed to investigate the surface of the membrane. To quantitatively analyze the number and size of pores on the surface, SEM images of each membrane (250 × 250 µm^2^) were analyzed using the Image J program (Image Processing and Analysis in Java 1.8.0_172, National Institutes of Health, Maryland, USA).

## 3. Results and Discussions

**Figure 2:**
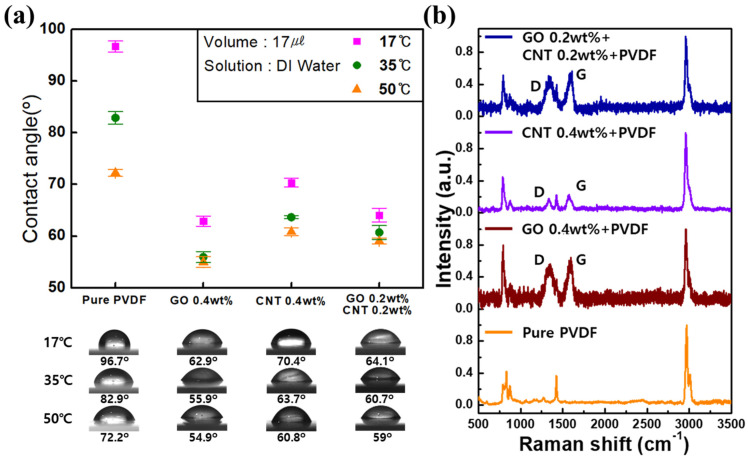
(**a**) Water contact angles of each PVDF membrane with changes in the content of GO and MWCNTs and coagulation temperature. (**b**) Raman spectra for each GO and MWCNT content.

As shown in Figure 2a, the water contact angles were measured five times at 17 °C for pure PVDF (96.7 ± 1.08), 0.4 wt% GO (62.9 ± 0.99), 0.4 wt% CNTs (70.4 ± 0.84), and 0.2 wt% GO + 0.2 wt% CNTs (64.1 ± 1.33). For each membrane the water contact angles at 35 °C were 82.9 ± 1.21 (pure PVDF), 55.9 ± 1.07 (0.4 wt% GO), 63.7 ± 0.30 (0.4 wt% CNTs), and 60.7 ± 1.32 (0.2 wt% GO + 0.2 wt% CNTs); and at 50 °C were 72.2 ± 0.70 (pure PVDF), 54.9 ± 1.04 (0.4 wt% GO), 60.8 ± 0.72 (0.4 wt% CNTs), and 59 ± 0.60 (0.2 wt% GO + 0.2 wt% CNTs).

In all the conditions, the water contact angles for pure PVDF were higher than those of the PVDF membranes with GO and MWCNTs. This was attributed to the hydrophobic characteristics of pure PVDF. In the case of membrane with added GO/MWCNTs, it was confirmed that the hydrophobic characteristics of the membrane changed to hydrophilic characteristics owing to the influence of oxygen and hydrophilic functional groups such as amine. Additionally, coagulation temperatures exhibited a significant effect on the surface energy, which determined the hydrophobicity or hydrophilicity of the PVDF membranes. As the coagulation temperature increased, the hydrophobic properties of the surface were hydrophilically modified, and this effect was observed to be the greatest in pure PVDF. These results indicated that the non-solvent temperature affected the properties of the membrane.

Raman spectroscopy is a powerful method for characterizing carbon materials [35,36,37]. Therefore, we observed Raman spectra to confirm the presence of GO and MWCNTs in the PVDF membrane, as shown in Figure 2b. The peaks of PVDF were observed at 840 and 513 cm^−1^, which corresponded to the CF_2_ bending vibration and out-of-phase combination of the CH_2_ rocking and CF_2_ stretching modes, respectively [38]. Additionally, in case of the addition of GO and MWCNTs, a G band partial peak corresponding to the $sp^{2}$ bond of carbon and a D peak attributed to the presence of disorder in carbon materials were confirmed at 1590 and 1350 cm^−1^, respectively. The Raman spectra indicated that the GO and MWCNTs were well distributed in the PVDF membrane.

**Figure 3:**
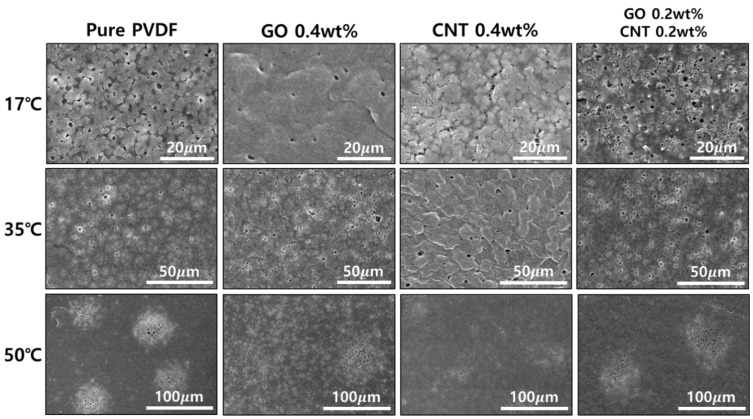
FE-SEM images of the surface of PVDF membranes according to various GO and MWCNT contents and coagulation temperatures, respectively.

The FE-SEM images in Figure 3 show the surface modifications based on the content of GO/MWCNTs and the coagulation temperatures of non-solvents (i.e., 17, 35, and 50 °C). Pores on the surface were generated during surface coagulation in the phase transfer process of the PVDF.

Pores on the front and rear surfaces of the PVDF membrane were present in an asymmetric structure, which resulted from the polymer concentration gradient when the membranes were immersed in a non-solvent coagulation bath [39]. Additionally, it was confirmed that the pores were formed differently in terms of morphology, diameter, and number of pores according to each condition. However, after the addition of GO and MWCNTs to pure PVDF, there was a difference in the pore size and number of surface pores. Moreover, even with the same content of GO and MWCNTs, the shape of the cluster and number of pores changed significantly according to the coagulation temperature of the non-solvent. As shown in Figure 2, with the increase in non-solvent temperature it was observed that the pores formed clusters instead of showing a uniform distribution across the surface.

**Figure 4:**
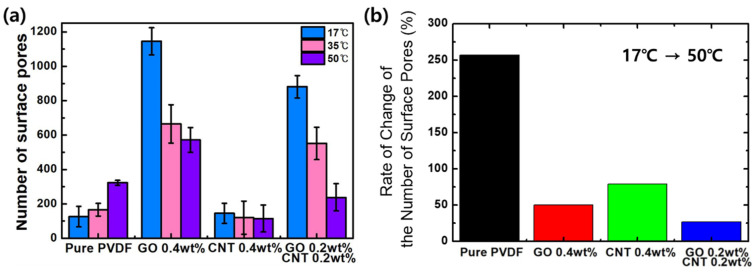
(**a**) The number of surface pores according to the content of carbon materials with changes in coagulation temperature. (**b**) Rate of change of the number of surface pores when the temperature increased from 17 to 50 °C.

Figure 4 shows the number and size of pores on each membrane analyzed using the Image J program. In the case of the pure PVDF, the number of pores increased from 126 ± 59.8 to 166 ± 36.8 and 323 ± 14.2 when the temperature increased from 17 to 35 and 50 °C, respectively. These results show that the number of pores increased by 256% at 50 °C as compared to 17 °C in pure PVDF (Figure 4b). Additionally, the numbers of pores in GO-PVDF were 1145 ± 79.6 at 17 °C, 665 ± 111.7 at 35 °C, and 572 ± 72.3 at 50 °C. As compared to pure PVDF, the number of pores in GO–PVDF significantly increased by 908% at 17 °C, 400% at 35 °C, and 178% at 50 °C. In contrast, in the same condition of GO samples, when the temperature increased the number of pores decreased to about 50%. This phenomenon was also observed in PVDF membranes with MWCNTs. As the temperature increased, the number of pores decreased by 21% from 145 ± 57.3 (17 °C) to 115 ± 78.5 (50 °C), with a large margin of error for each area. In addition, the number of pores at 17 °C were about 15% higher than those in pure PVDF. However, the number of pores in PVDF increased with the increase in the temperature. For PVDF membranes with added GO and MWCNTs, the number of pores decreased by 27% from 881 ± 64.5 at 17 °C to 238 ± 79.1 at 50 °C. In contrast to pure PVDF, the number of pores increased by 700% at 17 °C. However, the number of pores in PVDF membranes with added GO and MWCNTs was less than that of pure PVDF at 50 °C. Thus, the number and type of functional groups on the GO surface decreased in inverse proportion to increased coagulation temperature [40], which induced the decrease in the diffusion rate of the solvent and non-solvent and subsequently reduced the number of surface pores for the PVDF samples with GO. However, further research is needed on the detailed mechanism of interaction between GO and MWCNTs, which showed the lowest rate of change of the number of surface pores for the sample of GO 0.2wt% + CNT 0.2 wt%.

**Figure 5:**
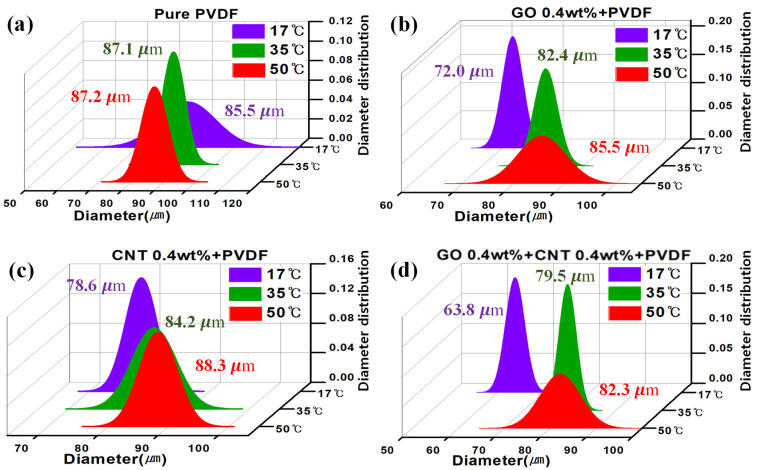
Diameter distribution of pores of membranes at different coagulation temperatures with (**a**) pure PVDF, (**b**) 0.4 wt% GO, (**c**) 0.4 wt% CNT, and (**d**) 0.4 wt% GO + 0.4 wt% CNT, respectively.

In addition, the diameter of the pores was analyzed, and the distribution plots are shown in Figure 5. In the case of pure PVDF, no significant change was observed in the pore size when the temperature increased from 17 °C (85.4 ± 8.6 μm) to 50 °C (87.3 ± 4.09 μm). Contrarily, the pore size increased by 18% from 72.1 ± 2.02 μm at 17 °C to 85.5 ± 4.74 μm at 50 °C for the GO–PVDF membrane. Similar trend was observed for the MWCNT–PVDF membrane. The pore size increased by 12% from 78.6 ± 2.61 μm at 17 °C to 88.3 ± 3.14 μm at 50 °C. This characteristic was further strengthened for the PVDF samples mixed with both GO and MWCNTs. It increased by 29% from 63.8 ± 2.08 μm at 17 °C to 82.3 ± 4.43 μm at 50 °C. Although the distribution of pore diameters varied by experimental conditions, it was found that the pore diameter was overall distributed between approximately 65 μm and 95 μm. Contrarily, it was confirmed that when GO and MWCNTs were added to PVDF the absolute pore size decreased when compared to that of pure PVDF.

In addition to the analysis of the size and the number of surface pores, the cross-section images of each membrane were observed by FE-SEM, and the morphologies of the inside pores were investigated as shown in Figure S1.

As a result, it can be seen that the pore size in the cross-section increased as the temperature increased. In addition, similar to the results from analysis of the surface pores, a large number of pores in the cross-section were observed in the GO-based PVDF membrane. For the MWCNT-based PVDF membrane, longer-shaped pores were observed from the top surface to the bottom surface of the membrane. A similar phenomenon was observed for the GO/CNT-based PVDF membrane, and the number of pores increased compared to pure PVDF membrane, as shown in Figure S1.

These results can be analyzed as follows: when the PVDF membrane was manufactured using the NIPS method, various variables such as manufacturing solvent, process method, and temperature significantly affected the surface properties. The speed of the phase transition process based on the non-solvent coagulation temperature significantly affected the properties of the pores. Additionally, the porous morphology was governed by the solvent–nonsolvent diffusion rate and the solidification rate [25,41]. When the solvent–nonsolvent mutual diffusion rate was high, instantaneous demixing occurred along with the formation of a macrovoid structure. When the mutual diffusion rate was low enough to undergo delayed demixing, a bicontinous structure was observed [25,42,43,44,45,46,47].

**Figure 6:**
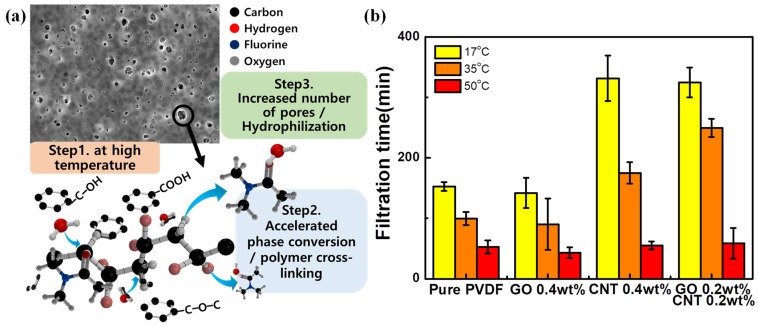
(**a**) The concept figure of pore modification process of carbon material-based PVDF membrane. (**b**) Filtration time of each PVDF membrane.

In the case of pure PVDF, the pore size was approximately same even when the temperature increased. However, for the PVDF membrane with GO and MWCNTs, the pore size increased with the increase in temperature. Additionally, the rate of change in the number of pores of the GO-based PVDF membrane showed a significantly larger value than that of the pure PVDF membrane. The increased temperature induced an increase in the diffusion rate of the solvent and the non-solvent [44,48]. As shown in Figure 6a, the effects of the functional groups of GO and MWCNTs and the high coagulation temperature can be explained. These results suggested that the hydrophilic functional groups of GO and MWCNTs accelerated the rate of phase conversion and contributed to the polymer cross-linking, which led to the solidification of membranes and eventually induced increase in the size and number of pores on the PVDF membrane [39], as shown in Figure 6. These results indicated that the surface properties of PVDF membrane can be modified by adding GO and MWCNTs to pure PVDF and changing the coagulation temperature.

Additionally, the water flux was measured to characterize the membrane properties according to each membrane condition, as shown in Figure 6b. This water flux was measured five times under each condition by vacuum filtration using 250 mL DI water at a pressure of 0.6 bar. The filtration time decreased with the increase in temperature from 17 °C to 50 °C, under all conditions. These results were attributed to the increased pore size of the membrane because of the increase in temperature. Particularly, in case of pure PVDF, with the increase in non-solvent temperature the pore size did not change significantly; however, the filtration time decreased owing to the increase in the number of surface pores. Although the pore size was slightly smaller, the PVDF with GO exhibited a shorter filtration time compared to that of pure PVDF, owing to the significant increase in number of pores. The PVDF with MWCNTs exhibited a significant filtration time, because the pore size was smaller than that of the pure PVDF membrane.

## 4. Conclusions

In this study, GO and MWCNTs were added to a pure PVDF membrane, and the surface characteristics and structural changes in the nanopore were investigated with the changes in coagulation bath temperature from 17 to 50 °C. We observed the Raman spectra, water contact angles, and FE-SEM images of the membranes. It was confirmed that GO and MWCNTs were well distributed in the PVDF membrane. The results of the water contact angle measurements showed that the hydrophobic properties of pure PVDF were hydrophilically modified by adding carbon materials. Additionally, according to the carbon content and temperature the number of pores, their rate of change, and pore sizes were analyzed using FE-SEM analysis. In addition, the water flux characteristics of pure PVDF and carbon material-based PVDF were comprehensively analyzed by filtration time analysis. In other words, the hydrophilic functional groups of carbon materials accelerated the rate of phase conversion and increased the coagulation bath temperature, which further increased the diffusion rate of the solvent and non-solvent. Therefore, it was confirmed that the introduction of the GO/MWCNT materials to the pure PVDF membrane and the adjustment of coagulation bath temperature improved the surface properties and modified the nanopore structures. These results can accelerate the convergent research on carbon materials and PVDF membranes, for example regarding highly increased water treatment. In addition, further research is needed on how these improved properties and the various functional groups of the carbon material affect water treatment performance.

## Data Availability

Not applicable.

## Supplementary Materials

The following are available online at https://www.mdpi.com/article/10.3390/nano11092269/s1, Figure S1: FE-SEM images of the cross section of membranes according to coagulation bath temperature with (a) pure PVDF, (b) 0.4 wt% GO, (c) 0.4 wt% MWCNTs, and (d) 0.4 wt% GO + 0.4 wt% MWCNTs, with scale bar (200 μm), respectively, Section S1: Information about the dimensions and chemical composition of GO and CNTs, Section S2: Brand and model of the equipment of the water contact angle measurement, Figure S2: Photo of the contact angle measurement equipment.
