# Supplementary material for: Modification of the Surface Morphology and Properties of Graphene Oxide and Multi-Walled Carbon Nanotube-Based Polyvinylidene Fluoride Membranes According to Changes in Non-Solvent Temperature
Source: Nanomaterials (Basel). 2021 Aug 31;11(9):2269. doi: 10.3390/nano11092269 (PMC8464745; doi:10.3390/nano11092269)
Supplement: Supplementary file 1 [file nanomaterials-11-02269-s001.zip › nanomaterials-1342371-supplementary.pdf]

nanomaterials

MDPI

Article

Modification of the Surface Morphology and Properties of Graphene Oxide and Multi-Walled Carbon Nanotube-Based Polyvinylidene Fluoride Membranes According to Changes in Non-Solvent Temperature

Jungryeong Chae †, Taeuk Lim †, Hao Cheng † and Wonsuk Jung *

School of Mechanical Engineering, Chungnam National University, Daejeon 34134, Korea; wndfud486@naver.com (J.C.); taewook9409@g.cnu.ac.kr (T.L.); chenghao@g.cnu.ac.kr (H.C.)
* Correspondence: wonsuk81@cnu.ac.kr; Tel.: +82-42-821-6647
† Contributed equally to this work.

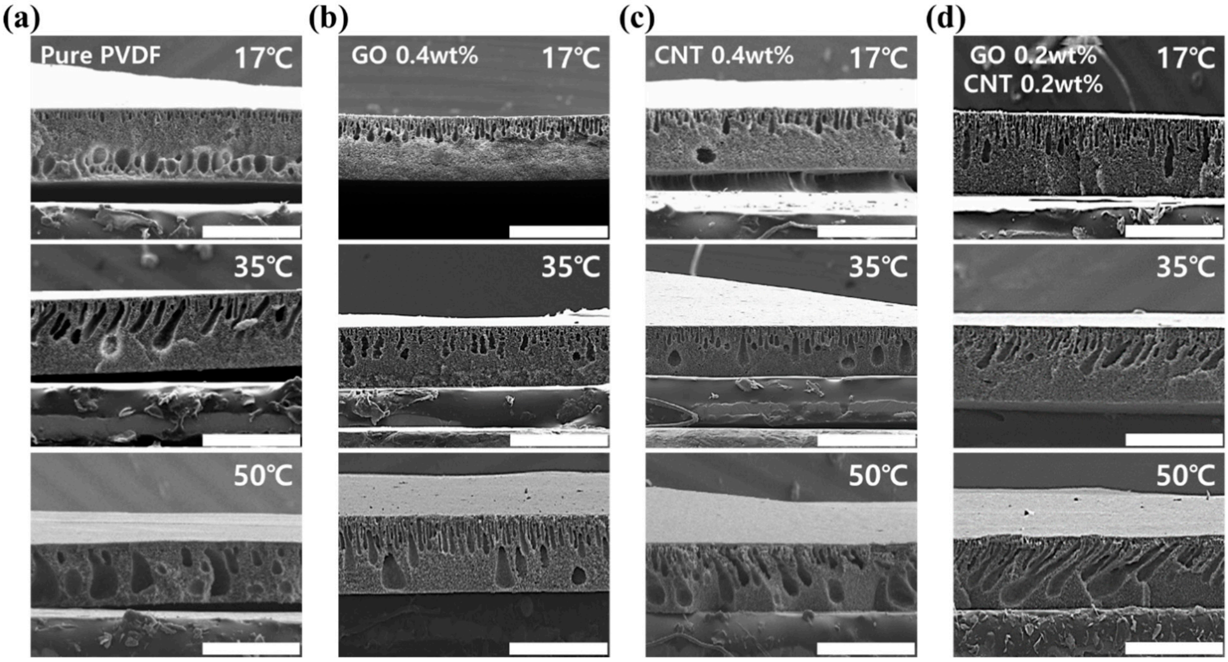

Figure S1. FE-SEM images of the cross section of membranes according to coagulation bath temperature with (**a**) pure PVDF, (**b**) 0.4 wt% GO, (**c**) 0.4 wt% MWCNTs, and (**d**) 0.4 wt% GO + 0.4 wt% MWCNTs, with scale bar (200 μm), respectively.

1. Information about the dimensions and chemical composition of GO and CNTs

1. *The properties of GO*

V-20, particle size: 18-104 μm (D_{50}: 50 μm), thickness: 1.1-1.3 nm, carbon: 45-55 %, oxygen: 35-45%, hydrogen ≤ 5%, sulfur ≤ 5%, nitrogen ≤ 1%.

2. *The properties of CNTs*

CM-130, outer diamteter ranged: 10-15 nm, aspect ratio: 2×10^3, purity > 90wt%, density: 0.05 g/cm^3.

2. Brand and model of the equipment of the water contact angle measurement

1. *Model Name*

   Phoenix-10

2. *Company*

   Surface Electro Optics

3. *Photo Image*

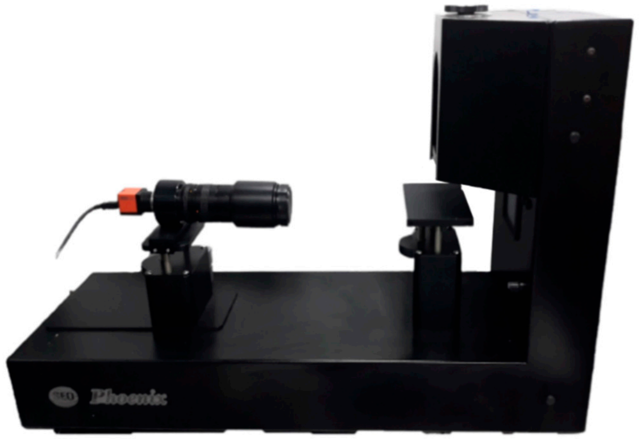

Figure S2. Photo of the contact angle measurement equipment.
